# Cluster-Based Analysis of Retinitis Pigmentosa Modifiers Using *Drosophila* Eye Size and Gene Expression Data

**DOI:** 10.3390/genes13020386

**Published:** 2022-02-21

**Authors:** James Amstutz, Amal Khalifa, Rebecca Palu, Kaushara Jahan

**Affiliations:** Department of Biological Sciences, Purdue University Fort Wayne, Fort Wayne, IN 46805, USA; james.m.amstutz@gmail.com (J.A.); palur@pfw.edu (R.P.); jahak01@pfw.edu (K.J.)

**Keywords:** retinal apoptosis, Endoplasmic reticulum (ER) stress, K-Means clustering, modifier genes, gene expression, phenotypic variation, degenerative models

## Abstract

The goal of this research is to computationally identify candidate modifiers for retinitis pigmentosa (RP), a group of rare genetic disorders that trigger the cellular degeneration of retinal tissue. RP being subject to phenotypic variation complicates diagnosis and treatment of the disease. In a previous study, modifiers of RP were identified by an association between genetic variation in the DNA sequence and variation in eye size in a well-characterized *Drosophila* model of RP. This study will instead focus on RNA expression data to identify candidate modifier genes whose expression is correlated with phenotypic variation in eye size. The proposed approach uses the K-Means algorithm to cluster 171 *Drosophila* strains based on their expression profiles for 18,140 genes in adult females. This algorithm is designed to investigate the correlation between *Drosophila* eye size and genetic expression and gather suspect genes from clusters with abnormally large or small eyes. The clustering algorithm was implemented using the R scripting language and successfully identified 10 suspected candidate modifiers for RP. This analysis was followed by a validation study that tested seven candidate modifiers and found that the loss of five of them significantly altered the degeneration phenotype and thus can be labeled as a *bona fide* modifier of disease.

## 1. Introduction

Retinitis pigmentosa (RP) is a degenerative disease resulting in the death of cells in the retina—the light-sensitive tissue that lines the back of the eye [1]. The disorder affects 1 in 4000 people worldwide, beginning with night blindness and tunnel vision and often leading to a complete loss of vision [2]. Approximately 15–35% of RP cases are inherited in an autosomal-dominant (AD) manner, meaning that a single copy of the causative mutation triggers the disease [2]. In addition, approximately 25–30% of the AD-inherited RP cases are found in the gene *rhodopsin* (*RHO*) [3]. These mutations commonly lead to misfiling proteins, endoplasmic reticulum stress and apoptosis [4].

Chow et al. analyzed the genetic factors that influence RP progression using the *Drosophila* Genetic Reference Panel (DGRP) [5]. The DGRP is a collection of ~200 inbred *Drosophila* strains that capture natural variation that exists in a wild population [6]. All strains are whole genome sequenced so that the identity of every base of the genome in each strain is known. This tool enables the exploration of how diseases and pathways are impacted by genetic variations, as well as whether these findings can be applied to humans.

Chow et al.’s study used the DGRP genomic sequence data to identify correlations between genetic and phenotypic variation in *Drosophila* eye size for Ryoo et al.’s model of RP, in which a mutated version of *rhodopsin* is overexpressed in the eye, leading to the accumulation of misfolded proteins and ultimately cell death [7]. As part of their experiment, Chow et al. highlighted the overexpression of a mutated version of the *RHO* gene, Rh1^G69D^. To determine the impact of variation on retinal degeneration, Chow et al. crossed this model to 173 DGRP strains and measured their mean eye sizes as a proxy for the degree of cell death [5]. They then performed an association analysis to link existing genetic variation in the DGRP to eye size variation. More than 100 candidate genes were identified, nearly 80% of which have a conserved human orthologue [5]. Several of these have already been shown to influence the degree of degeneration in the RP model, validating this approach [5,8,9].

However, this method cannot account for potential modifiers whose expression is regulated in *trans* to the variation in the DNA sequence. These modifiers may be differentially regulated several steps downstream of the associated gene or be too far from the regulatory element to have been identified in the original study. They would thus require an examination of their differential expression on the strains of the DGRP. 

This kind of datum was collected by Huang et al. when they examined the widespread sexual dimorphism and modularized expression patterns for *Drosophila* to characterize its level of transcriptome diversity [10]. Using the DGRP as a basis for quantitative trait loci (QTL) mapping, their research produced gene expression data for adult male and female *Drosophila*. Interestingly, at least one of the genes identified as a modifier of RP in the previous study was shown to have differential expression that correlated with the degree of degeneration in the Rh1^G69D^ protein [8]. This suggests that a subset of modifier genes may be identifiable by observing differences in gene expression that can be correlated with eye size.

The objective of the research reported in this paper is to computationally identify candidate modifiers for RP using the DGRP expression data. Our foundational datasets are the eye sizes associated with the Rh1^G69D^ model in the DGRP strains, as identified in Chow et al. 2016 [5], and the RNA expression values in Huang et al. 2015 [10]. With these datasets, we analyze the phenotypic outcomes of RP and find what genes from the collection vary in their expression in relation to eye size. A K-Means clustering algorithm is used to gather candidate genes that influence RP by investigating the correlation between the RNA expression values and eye sizes of diseased *Drosophila* strains.

The rest of this paper is dedicated to the background, methodology, results and conclusions drawn for a proposed K-Means-based clustering algorithm to identify RP candidate genes. First, related work on RNA sequencing, differential gene expression (DGE) analysis and clustering will be discussed. The structure of the Rh1^G69D^ and the DGRP datasets and the steps taken to calculate their correlation coefficients will also be detailed alongside the algorithm’s filtering method. Next, the resulting list of suspected candidate modifier genes will be presented, analyzed and validated to determine which genes have the strongest ties to RP. Finally, future directions that could expand the short and long-term scope of this research are discussed.

## 2. Gene Expression Data Analysis

Differential gene expression (DGE) analysis is an important step in the RNA-seq pipeline. DGE analysis identifies which genes are expressed at different levels between conditions, providing insight into the biological processes affected by changes in such condition(s) [11]. A comparative study by Soneson and Delorenzi highlights several algorithmic approaches to identify phenotypic variation [12]. They developed eleven methods of DGE analysis in the R scripting language using simulated and real RNA sequences to determine which ones best identify genes whose change in expression values is statistically significant. These methods were implemented and evaluated on 12,500 synthetic and 11,870 real genes from their respective two datasets. The results show that voom + limma and vst + limma are computationally fast and transformation based. DESeq, on the other hand, proved to be the most conservative of the methods providing nominal *p*-values, while TSPM and EBSeq had the strongest sample size. Soneson and Delorenzi concluded that there were pros and cons to each of these methods, with an overarching negative being the small sample size of RNA sequences, but the voom + limma and vst + limma methods performed best in gathering genes under multiple conditions. A more recent comparative study by Wang et al. [13] discussed eight methods for DGE analysis of single-cell RNA-seq (scRNAseq) data. 

Clustering is also an important tool for analyzing gene expression data. The goal of clustering is to identify groups that are aggregated together because of certain similarity, where members of the same clusters are more similar in some way to each other than to members of other clusters. Applying this to RNA-seq data means identifying clusters of genes that exhibit similar expression profiles across samples indicating a particular macroscopic phenotype, such as cancer [14,15].

## 3. Materials and Methods

### 3.1. Data Description

The dataset contributions from Chow et al. [5] and Huang et al. [10] are formatted as text files. Table 1 shows the first fifteen rows of the Rh1^G69D^ file. The data are organized in two columns, one for strain names and the other for mean eye sizes. The range of mean eye sizes is from 14,254.60 to 27,349.11 and are measured in pixels × 10^3^. The Rh1^G69D^ file contains 173 strains. On the other hand, the DGRP file stores the gene expression data for 184 strains over 18,140 genes. Table 2 shows only the first ten rows and first five columns of the dataset. It is a matrix where the rows represent gene IDs and the columns represent the *Drosophila* strains/lines. The values stored in the cells of the matrix represent the genetic expression values of each strain for each gene. It is worth noting that the expression values were originally measured and collected for two replicates of each strain. For example, the gene FBgn0000014 has two columns annotated as line_21:1 and line_21:2 with expression values of 4.245 and 4.216, respectively. There is also a distinction between gene ID prefixes in the DGRP data; those with the FBgn prefix are typically annotated online, whereas little is known about those with the XLOC prefix. Furthermore, the DGRP dataset contains 184 strains, of which only 171 intersect with the strains represented in the Rh1^G69D^ dataset. Therefore, those 171 strains will be the focus of our analysis.

### 3.2. Data Clustering Methodology

K-Means clustering is a typical clustering method used in the field of DGE analysis. K-Means belongs to a category of unsupervised learning algorithms since it can group multidimensional datasets without referring to a known outcome. The algorithm attempts to divide *m* points in *n* dimensions into *k* clusters to minimize the sum of the squares within the clusters [16]. This means that the number of clusters *k* needs to be defined *a priori.* The algorithm proceeds by finding a *centroid* for each cluster that *m* points groups around according to the distance between the other centroids [17]. Once all points have been assigned to a cluster, the positions of the *k* centroids are recalculated until they exhibit little to no change. Let *x* = {*x*_1_, *x*_2_, …, *x_m_*} be the set of data points and *v =* {*v*_1_, *v*_2_, …, *v_k_*} be the set of centers. The pseudocode for K-Means clustering is as follows, where *c_i_* refers to the number of data points in the *i*th cluster and *k* represents the number of cluster centers [18]:

1. Randomly select *k* cluster centers;
2. Calculate the distance between each data point and all cluster centers;
3. Assign the data point to the cluster whose distance from the center is minimum of all cluster centers;
4. Recalculate the new cluster centers using

(1) $$v_{i}=\left(\frac{1}{c_{i}}\right)\overset{c_{i}}{\underset{j=1}{\sum }}x_{j}$$

5. Recalculate the distance between each data point and new obtained cluster centers;
6. If no data point was reassigned, stop; otherwise, repeat from step 3.

**Figure 1:**
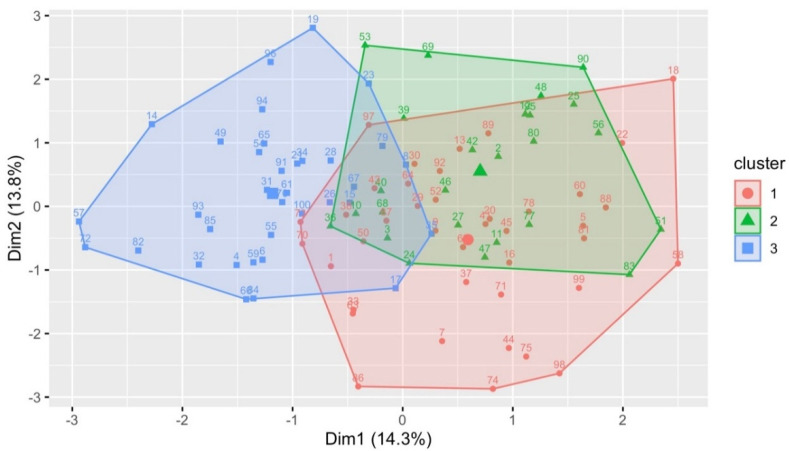
K-Means clustering example for three clusters.

Figure 1 illustrates a color-coded result for three clusters of 2D points using the steps above.

**Figure 2:**
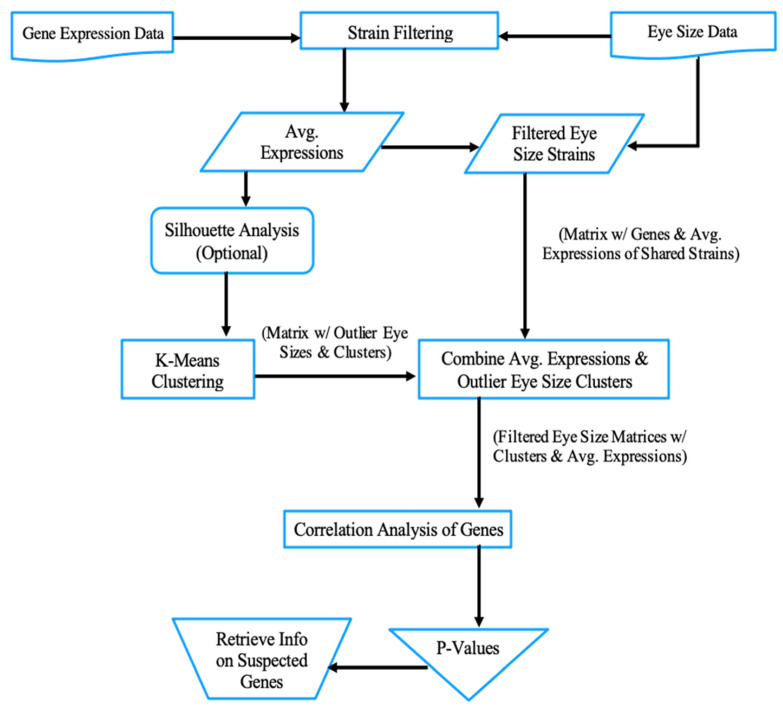
The proposed K-Means clustering algorithm based on gene expression data.

Since the K-Means algorithm specializes in iteratively categorizing data, it can be used to guide the process of discovering candidate modifiers for RP. So, the main objective of the clustering process in this study is to group the strains according to their expression values of different genes. The main steps of the proposed algorithm are illustrated in Figure 2. The algorithm starts with reading the datasets of the Rh1^G69D^ and the DGRP expression from the files. The datasets are loaded into two matrices that next undergo a filtering step to make sure that only those strains featured in both datasets are kept. Considering only the filtered strains, the expression values of the two annotated DGRP lines are averaged. For example, line_21:1 and line_21:2 for the gene FBgn0000014 from Table 2 are averaged into one expression value of 4.231. Then, the K-Means clustering step works on the average genetic expressions of all filtered strains. The averaged expressions can optionally be tested with silhouette analysis prior to undergoing K-Means clustering. Silhouette analysis measures the tightness and separation of a given cluster compared to its neighbors and evaluates the appropriate number of clusters based on proximity [19].

The identified clusters actually group the strains based on their genetic expression profile. In other words, one of the clusters is expected to contain a high number of exclusively minimum expression values over some specific genes and vice versa. The two clusters exhibiting minimum or maximum gene expression profiles are categorized, in this research, as outlier clusters. The matrix of averaged expressions for the outlier clusters are carried over the next step and merged with the filtered eye size strains based on strain numbers. In fact, this will help further categorize the outlier clusters based on their average eye sizes. More specifically, to determine the clusters representing outlier eye size grouping, the range of eye sizes is partitioned into 4 quadrants by subtracting the maximum and minimum eye size values and dividing the result by 4. The upper threshold is set one quadrant less than the maximum eye size, while the lower threshold is set one quadrant from the minimum eye size. Hence, the strains of the outlier clusters will have their mean eye sizes compared against these thresholds to see how many strains per cluster go above or below the upper and lower threshold values. The cluster with more strains with eye sizes exceeding the upper threshold or less than the lower threshold will be considered an outlier cluster with the highest or lowest mean eye sizes, respectively. With this done, the next step will be focusing on analyzing the correlation between the mean eye size and the genes for the outlier clusters. 

### 3.3. Correlation Analysis

Correlation coefficients statistically assess the correlation between two quantitative variable datasets by calculating their covariance. We will be primarily using Pearson’s correlation coefficient *r*, whose value lies between −1 and 1. With these variables denoted as *z_x_* and *z_y_*, the two can be considered correlated if some values associated with one variable tend to occur more often with some values of the second [20]. So, if *r*’s value is close to zero *z_x_* and *z_y_* have a weak correlation, and if close to −1 or 1 they have a strong negative or positive relationship, respectively [21]. The formula for *r* is:

(2) $$r=\frac{\sum z_{x}z_{y}}{n-1}$$

where *n* is the number of observations [22]. In this research, *z_x_* and *z_y_* represent the filtered mean eye sizes and averaged expression values for a specific gene, respectively. Therefore, if a gene gathered from the outlier K-Means clusters exhibits a strong association, it can be considered a possible candidate modifier for RP. The *p*-values of these genes will also be calculated to assess any correlation’s significance against the null hypothesis, a suggestion that no statistical relationship exists between the two sets of data. The null hypothesis tested in this research is that the average gene expression is equal across all groups (i.e., the gene is not differentially expressed), and this hypothesis will be rejected if *z_x_* and *z_y_* demonstrate significant different expression distributions (i.e., the gene is differentially expressed). Hence, any gene exhibiting a correlation coefficient value nearing −1 or 1 and having a *p*-value below 5% (0.05) will be considered statistically significant.

Although Pearson’s formula is the most commonly used correlation method, Kendall and Spearman’s coefficients will also factor into the post-clustering analysis for this research. Kendall’s formula for τ analyzes the concordance and discordance of its paired observations. It is computed as follows [23]:

(3) $$\tau =\frac{n_{c}-n_{d}}{n_{0}}$$

where *n* is the sample size, *n*_0_ the unique unordered pairs of observations, *n_c_* the number of concordant pairs and *n_d_* the number of discordant pairs, or *n*(*n −* 1)/2. Concordant pairs are sets of data that increase and decrease in a way that signifies a relationship whereas discordant pairs demonstrate no such patterns. A value of 1 for Kendall’s τ means a perfect relationship for the dataset exists and 0 means no relationship exists. Negative values approaching −1 can also exist for τ, but unlike the other Pearson’s correlation, this is no different from a positive value approaching 1.

On the other hand, Spearman’s coefficient, *r_s_*, tests the strength of a linear relationship between two quantitative variables by emphasizing ordinal associations and direction [23]. The formula for *r_s_* analyzes the ranked data for *X_i_* and *Y_i_* as follows:

(4) $$r_{s}=\frac{\sum_{i=1}^{n}\left\{\left(x_{i}-\bar{x}\right)\left(y_{i}-\bar{y}\right)\right\}}{\sqrt{\sum_{i=1}^{n}{\left(x_{i}-\bar{x}\right)}^{2}}\sqrt{\sum_{i=1}^{n}{\left(y_{i}-\bar{y}\right)}^{2}}}$$

where *n* is the sample size, *x_i_* is the rank of the measurement of *X* taken on the *i*th individual, and *y_i_* is the same for *Y*. $\bar{x}$ and $\bar{y}$ can be further defined as:

(5) $$\bar{x}=\frac{1}{n}\sum_{i=1}^{n}x_{i},\;\bar{y}=\frac{1}{n}\sum_{i=1}^{n}y_{i},$$

The value of Spearman’s *r_s_* ranges identically from −1 to 1. Furthermore, both Kendall’s coefficient τ and Spearman’s coefficient *r_s_* focus on broader monotonic relationships [23].

As an example of Pearson’s correlation coefficient, to calculate *r* for the gene FBgn0026250 *n*, *z_x_* and *z_y_* need to be known. *n* is the number of strains being observed for this iteration of calculating *r*, which in this case will be 171. *z_x_* is a vector of the 171 mean eye sizes being observed, and *z_y_* represents the expression values of the gene FBgn26250 for the 171 observed strains. The first three values in *z_x_* are 19,976.8, 21,473.2 and 19,981.5 pixels for strains 21, 26 and 38, respectively, as listed in Table 1, while the first three values in *z_y_* are 10.825, 10.867 and 11.000 for strains 21, 26 and 28, respectively. Applying Pearson’s formula for *r* on the values of *n, z_x_* and *z_y_* above, the correlation coefficient of FBgn0026250 is estimated to be −0.080. Since the value approaches 0, we can conclude that the expression of this gene is not strongly correlated with the degeneracy of the eye size. To contrast this, the correlation coefficient computed between the eye sizes and gene expression of the gene FBgn0026084 was found to be −0.229, indicating a stronger negative correlation. Furthermore, the gene FBgn0026064 showed a stronger positive correlation with *r* estimated to be 0.136. Notice that, for this study, *z_x_* is comparatively static and only changes based on the number of strains being correlated, whereas *z_y_* changes as each of the 18,170 genes are considered.

Using these same examples, Kendall’s correlation coefficient τ will be calculated for FBgn0026250, FBgn0026084 and FBgn0026064. The number of concordant pairs *n_c_* for FBgn0026250 is determined to be 6718 and the number of discordant pairs *n_d_* is 7817 after considering the vector for mean eye sizes and the gene’s expression values. In addition, the number of unordered pairs *n*_0_ is 14,535 if *n* remains 171, which results in τ’s value of −0.090. Applying the same steps for FBgn0026084 and FBgn0026064, τ is calculated as −0.169 and 0.094, respectively. Since FBgn0026084′s value of −0.169 is the farthest of the three sample genes from 0, it has the strongest correlation among them.

In the case of Spearman’s correlation coefficient *r_s_*, *x* represents the vector of mean eye sizes, *y* the expression for a given gene and *n* the sample size of 171. With $\bar{x}$ estimated to be 21,532.34 and $\bar{y}$ as 10.96146, Spearman’s correlation coefficient for FBgn0026250 is −0.094. When Spearman’s method is run for FBgn0026084 and FBgn0026064, *r_s_* is approximately −0.248 and 0.141, respectively. These three coefficient values are similar to Pearson’s −0.080, −0.229 and 0.136 for the same set of genes and thus suggest the same pattern of correlation. The primary difference between Pearson and Spearman’s correlation values for these examples is that the latter is less affected by outlier values in *x* and *y*.

### 3.4. Fly Stocks and Maintenance

Flies were raised at room temperature on a diet based on the Bloomington Stock Center standard medium with malt. The *GMR >* Rh1^G69D^ strain, which serves as the model of eye degeneration in this study, has been previously described [5]. Briefly, the *GMR-GAL4* transgenic driver promotes expression of a mutant, misfolded rhodopsin protein (Rh1^G69D^) through a second transgene (*UAS*-Rh1^G69D^) [5,7]. The following RNAi and control strains were crossed to the *GMR >* Rh1^G69D^ model for validation experiments and are from the Bloomington Stock Center: *Gycalpha99B* (64,009 and 28,748), *CG33177* (61,839), *Mnn1* (51,862, 31,220, and 35,150), *Ipk2* (60,081 and 35,255), *CG4558* (58,225), *Nedd8* (33,881), *CG4306* (65,890), control *attP40* (36,304), and control *attP2* (36,303).

### 3.5. Eye Imaging

For eye images, adult females were collected under CO_2_ anesthesia and aged to 2–7 days, then frozen. Eyes were imaged at 20× magnification using a Leica EZ4W stereo microscope and camera. Camera settings were as follows: Brightness 70%, γ 0.7, Saturation 106, Capture Format 2592 × 1944 pixels, Shading None, Sharpening Low. Flies were positioned to capture the left eye for all flies for consistency. In total, 10–15 images from individual flies were captured for each strain. Eye area was measured in ImageJ as previously described [5,8,9]. Briefly, the outlines of the eyes were carefully traced using the freeform drawing tool on ImageJ. Then, the two-dimensional area in pixels for the selection was calculated using ImageJ. This two-dimensional area is used as a proxy for three-dimensional eye size.

### 3.6. Statistics for Biological Validation

Statistics for comparisons between eye sizes of control versus RNAi strains were calculated using R software. *p*-values were determined using ANOVA with Dunnett’s multiple testing correction for eye size. A cutoff of *p* = 0.05 was used for significance.

## 4. Results

### 4.1. Experimental Setup

The proposed algorithm, shown in Figure 2, was implemented using the R scripting language. RStudio alongside external libraries were utilized to analyze the data from the aforementioned text files [24]. For example, ggplot2 and its subsidiary ggrepel package were used for creating data visualization. In addition, factoextra was used for developing K-Means plots [25,26].

**Figure 3:**
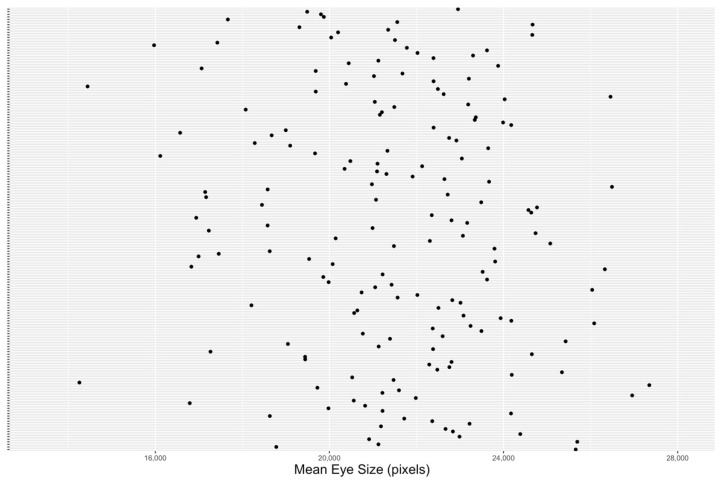
The distribution of Rh1^G69D^ mean eye sizes.

**Figure 4:**
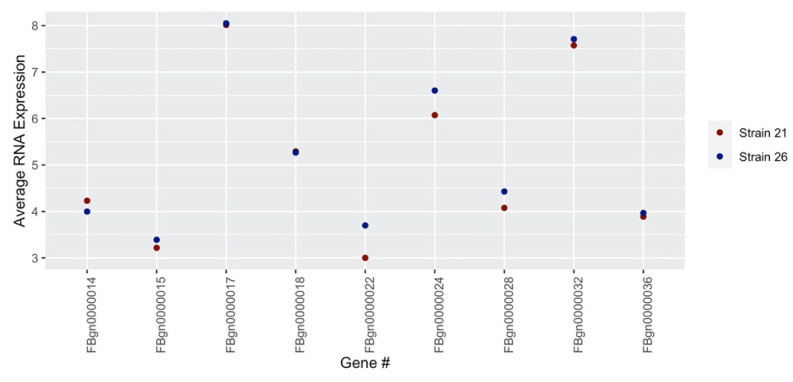
A Sample of averaged DGRP gene expression values for strains *s_21_* and *s_26_*.

The Rh1^G69D^ data, partly listed in Table 1, have 173 strains (e.g., RAL021, or *s*_21_) and just as many mean eye sizes. Figure 3 shows the distribution of the eye sizes for each strain, where the x-axis shows the strain numbers and the eye sizes (measured in pixels × 10^3^) are represented on the y-axis. The strain numbers are attached to the y-axis in the same order listed in the Rh1^G69D^ file. The DGRP expression data, partly listed in Table 2, have 369 columns documenting the names and annotated strains/lines for 18,140 genes. To show the complexity of the data, Figure 4 demonstrates the average expression values for the two strains and nine genes listed in Table 2. The x-axis represents the gene IDs in an incremental order and the y-axis represents the average expression values. Using *s*_21_ as an example, the mean expression values are calculated by averaging the gene expression values of line_21:1 and line_21:2 for every instance of these paired gene sets. It is worth noting that the strain numbers are used solely for the purpose of identification, and thus do not reflect any information about their respective eye sizes. For example, the final entry in the Rh1^G69D^ data, *s*_913_, does not have the highest or lowest mean eye size.

Furthermore, the data retrieved from the Rh1^G69D^ file needed to be filtered for multiple reasons. For example, strain *s_513_* was removed due to it missing line_513:1 in the DGRP expression file and its respective expression values, preventing it from being averaged. In addition, several strains were found to be exclusive to the Rh1^G69D^ or DGRP expression data, so they were excluded from the analysis as well. After all sets are cross-referenced with the list of 368 annotated DGRP lines, 171 strains are ultimately used.

### 4.2. Clustering Results

**Figure 5:**
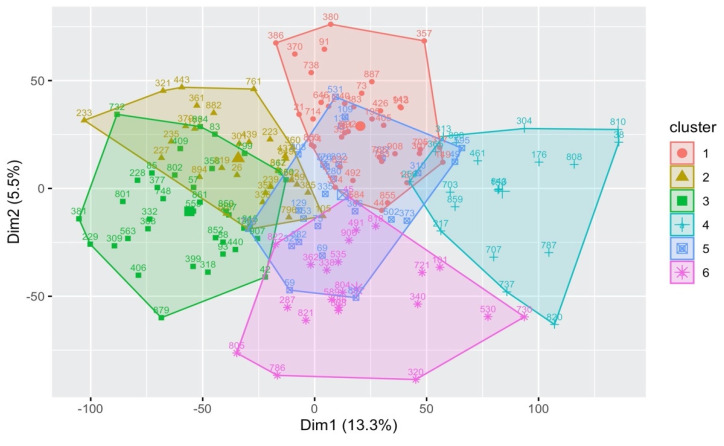
K-Means clusters for the DGRP genetic expression.

When used as a guide, silhouette analysis recommended producing two clusters due to the relatively low number of available strains. However, we found that two clusters are insufficient for identifying the outlier eye-size grouping. Therefore, the algorithm was tested for two to eight clusters, and we chose six clusters after noticing that a higher number of clusters reduced the coherence of the identified clusters. Figure 5 shows the K-Means clustering result for the 171 filtered strains based on their averaged genetic expressions. Cluster 1 and Cluster 3 were identified as the outlier clusters including 38 and 37 strains, respectively.

So, for the six clusters displayed in Figure 5, each node is labeled with its strain number, and the unlabeled larger nodes are the *centroids* of their clusters. The nodes in Figure 5 represent how closely matched each strain’s averaged expression values are to one another, and the x and y-axes are a measurement of this correlation instead of them being directly associated with eye size. In fact, the x-axis is given numerical values attributed to principal component analysis (PCA) that represent how a node’s average genetic expression compares to the others of its cluster. For example, *s*_38_ and *s*_810_ on the right edge of Cluster 4 nearly overlap, so they have similar expression values across all 18,140 genes. However, the outliers with the highest and lowest average eye sizes are difficult to identify here because PCA accounts for 18,140 dimensions (as many as there are genes), reducing linearity.

**Figure 6:**
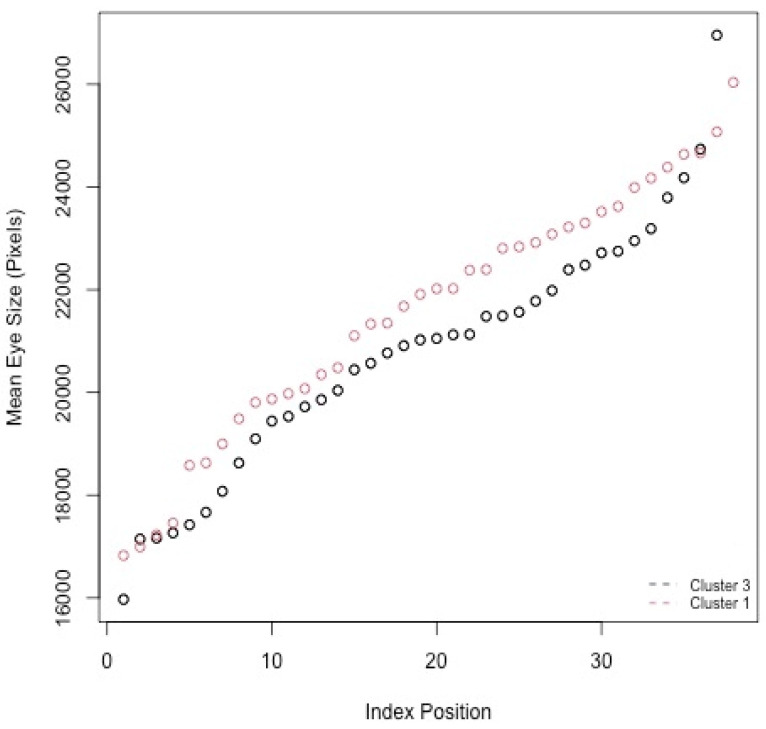
Rh1^G69D^ mean eye sizes for highest (Cluster 1) and lowest (Cluster 3) mean eye size grouping.

Therefore, to determine the clusters representing outlier eye size grouping, the upper threshold was selected as 24,075.48 (in pixels × 10^3^) and the lower threshold as 17,528.23. The partition value of 3273.628 was calculated based on the maximum and minimum eye size values of 27,349.11 and 14,254.6, respectively. The strains in Figure 5 then have their mean eye sizes compared against quadrants to see how many strains per cluster go above or below the upper and lower threshold values. Having more strains with eye sizes greater than 24,075.48, Cluster 1 was considered a representation of the highest eye size grouping. The opposite is true for Cluster 3, having more strains below 17,528.23 for the eye size, making it the probable outlier for the lowest eye size grouping. Figure 6 illustrates Cluster 1 and 3′s overlapping mean eye size measurements. The x axis represents the IDs of the strains as index positions. 

### 4.3. Correlation Results

Once the strains of Cluster 1 and Cluster 3 (from Figure 5) are identified as the outlier clusters, the next step involves calculating their correlation coefficients and *p*-values based on each individual gene’s expression. Notice that this analysis does not include all 171 filtered stains. Instead, it focuses only on the strains of the identified outlier clusters and how their eye sizes are correlated with the expression values of the 18,140 genes. The result of this analysis highlighted the top 20 genes with the highest absolute coefficient values and their *p*-values. This approach is repeated using the Pearson, Kendall and Spearman correlation tests, respectively. 

**Figure 7:**
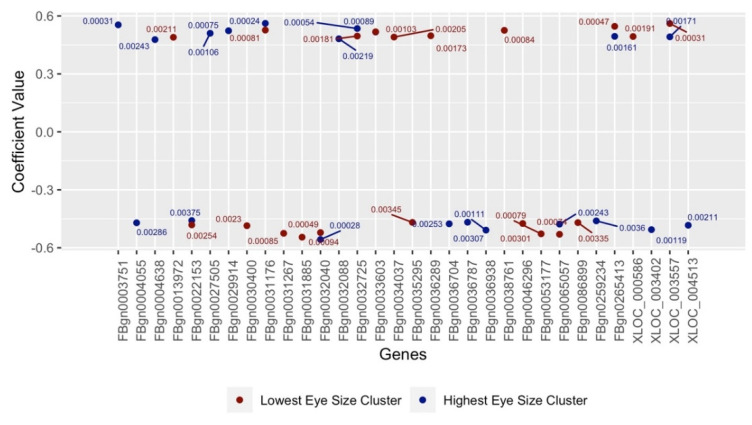
Suspect genes from Pearson expression clustering for outlier clusters.

**Figure 8:**
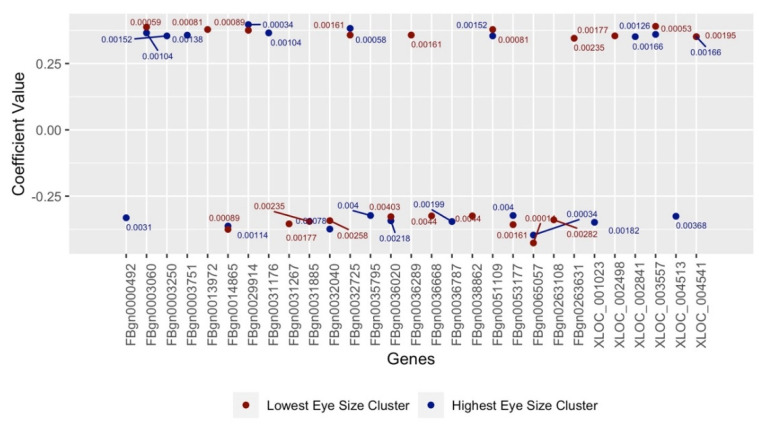
Suspect genes from Kendall expression clustering for outlier clusters.

**Figure 9:**
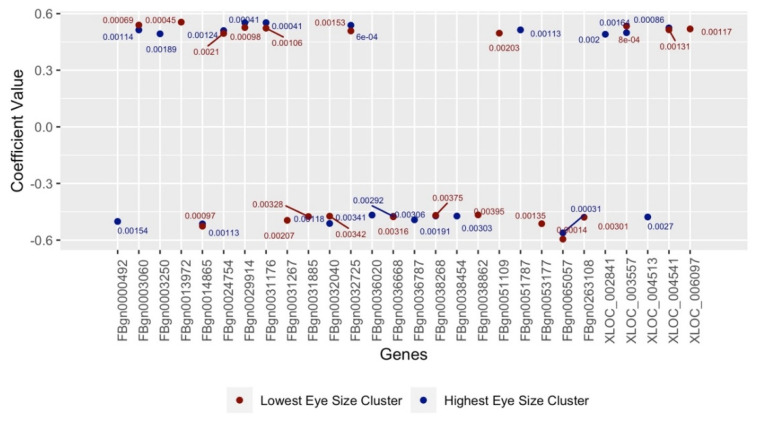
Suspect genes from Spearman expression clustering for outlier clusters.

The 20 genes gathered in each of Clusters 1 and 3 with the highest and lowest Pearson coefficient values are illustrated in Figure 7, with the plot’s values averaging ±0.4. Figure 7 also contains genes that represent a few of the highest and lowest coefficient values (and *p*-values) simultaneously. Figure 8 and Figure 9 differ from Figure 7 in that the Kendall and Spearman correlation methods were used, respectively, to calculate the coefficient values of suspected genes. According to these three Figures, the selected genes not only surpass the *p*-value significance percentage of <5% (0.05) but have correlation coefficients that average ± 0.5. This indicates a strong association between the highest/lowest mean eye sizes and averaged expressions values of the gathered genes. We found that 10 genes were shared among all three tests (Table 3) and thus considered the top candidate genes; hence, they were run through the candidate validation study.

**Figure 10:**
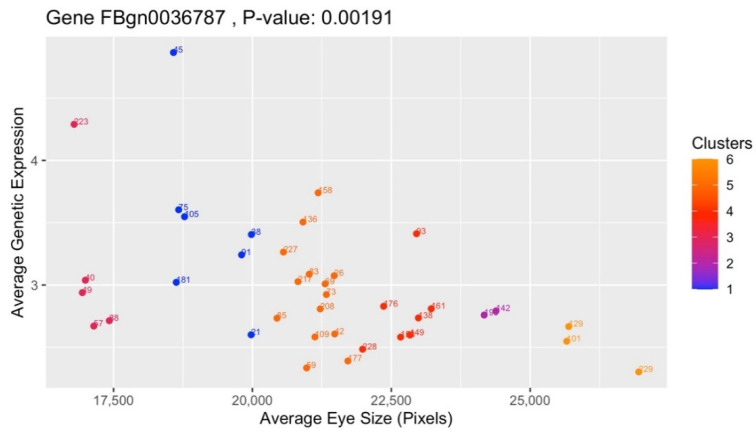
Gene FBgn0036787, from the highest mean eye size outlier cluster.

**Figure 11:**
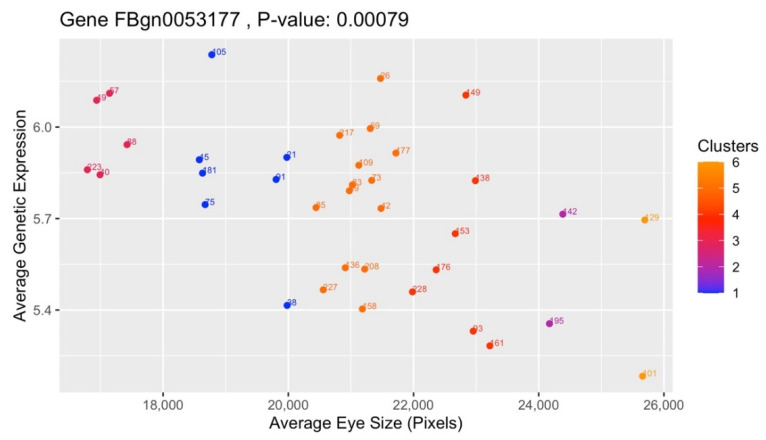
Gene FBgn0053177, from the lowest mean eye size outlier cluster.

One of the top candidates is the gene *CG4306* (FBgn0036787), which is plotted in Figure 10 using Pearson’s correlation method. It has a statistically significant *p*-value of 0.00307 due to it being <5%, or 0.05. The x-axis represents the average eye size of the strains (*z_x_*) and the y-axis FBgn0036787′s averaged genetic expression of the strains (*z_y_*). Although FBgn0036787 only features the 38 strains of Cluster 1, the gradients of the nodes shift between Figure 5′s six clusters due to *z_y_* factoring in all 171 strains into the calculation of *r*. Another candidate gene, *CG33177* (FBgn0053177), is depicted in Figure 11 and the 37 strains from Cluster 3 associated with it. It also has a statistically significant *p*-value of 0.00079.

### 4.4. Candidate Validation

**Figure 12:**
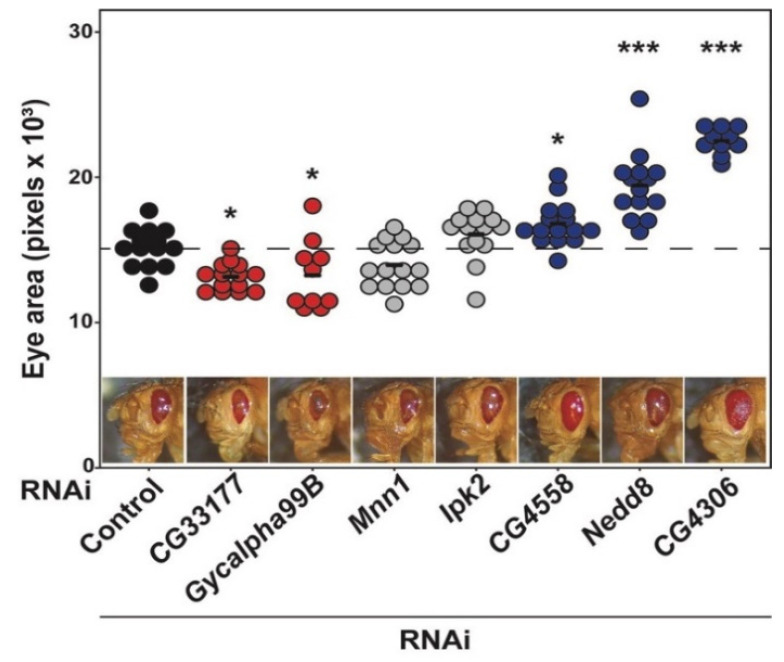
RNAi strains and the respective eye areas for suspected genes. *: *p*-value < 0.05; ***: *p*-value < 0.001.

To validate the candidate genes identified through this analysis (Table 3), we elected to test the impact of loss of modifier expression for seven candidate genes for which we were able to obtain transgenic RNAi lines. The RNAi transgene targets the candidate gene of interest, reducing or eliminating its expression in the target tissue, in this case the developing eye [27]. We crossed the RNAi strains targeting each of these modifiers into the *GMR >* RH1^G69D^ line, then measured the eye area in offspring carrying both the RNAi construct and the RP model, as shown in Figure 12.

Remarkably, we found that loss of five out of seven candidate modifiers significantly impacted eye size in the *GMR >* Rh1^G69D^ model of RP. Knockdown of either *CG33177* (13,153 ± 955 pixels, *N* = 14) or *Gycalpha99B* expression (13,259 ± 2385 pixels, *N* = 10) resulted in enhancement of the degenerative phenotype, showing a significant decrease in eye size compared to controls expressing only *GMR >* Rh1^G69D^ (15,136 ± 1347 pixels, *N* = 14) (Figure 12). Knockdown of *CG4558* (16,811 ± 1466 pixels, *N* = 15), *Nedd8* (19,439 ± 2286 pixels, *N* = 14), or *CG4306* (22,507 ± 916 pixels, *N* = 10) resulted in a partial rescue, with a significant increase in eye size compared to controls expressing only *GMR >* Rh1^G69D^ (Figure 12). No significant change in eye size was observed upon knockdown of *Mnn1* (13,967 ± 1612 pixels, *N* = 14) or *Ipk2* (16,066 ± 1694 pixels, *N* = 13) (Figure 12). These results were confirmed by independent RNAi lines for *Gycalpha99B*, *Mnn1*, and *Ipk2*, validating the ability of the proposed gene expression correlation analyses to identify *bona fide* modifiers of RP. 

## 5. Discussion

### 5.1. Suspected Candidate Modifiers

A total of 89 genes are featured in Figure 7, Figure 8 and Figure 9 after using this study’s K-Means algorithm to cluster for genetic expression and calculate any suspected gene’s Pearson, Kendall and Spearman correlation coefficients. Table 3 displays the 10 identified genes of these 89 that were run through candidate validation for being the most likely modifiers for RP. It lists a given gene’s annotated data, which includes their gene ID, symbol, name, human orthologue(s) and potential link to RP. We then validated the results of these ten genes and their significance in potentially contributing to RP. Several of these suspected genes (such as FBgn0032040 and FBgn0065057) also exhibited two sets of coefficient/*p*-values simultaneously across Figure 7, Figure 8 and Figure 9. This was likely made possible by this study’s algorithm keeping the strains of the outlier clusters separate without filtering out the expression values unassociated with these clusters.

### 5.2. Gene Annotation

Ultimately, the genes identified through this study are only candidate modifiers of the degeneration phenotype observed in these flies. They require validation and characterization of their impact on the RP model before they can be labeled as a *bona fide* modifier of disease. It is remarkable that of the seven tested candidate genes, five significantly altered the degeneration phenotype both quantitatively and qualitatively (Figure 12). The high positive percentage for these genes is likely due to the selection of only candidates shared across all three correlation analyses, and to the specificity of the eye size phenotype as a readout for ER stress and degeneration. 

These candidates are also interesting for their roles in pathways and processes related to RP. Two of the validated candidates (*CG33177* and *CG4558*) have links to oxidative stress, which activates several of the same pathways as ER stress. *CG33177* encodes a glutathione peroxidase that is orthologous to *MGST1* in humans [28]. The MGST1 enzyme localizes to the ER membrane, where it acts to protect from oxidative stress. Its reduced expression with age in the retinal pigment epithelium is thought to be linked to macular degeneration [29]. Loss of this gene in the fly RP model would likely be associated with increased sensitivity to stress, including the ER stress induced by accumulation of the misfolded Rh1^G69D^ protein. The result is the observed reduction in eye size that is suggestive of increased degeneration and cell death. *CG4558* is largely uncharacterized, but its closest human orthologue (*C6orf89*) encodes bombesin receptor-activated protein (BRAP), which interacts with the G-protein-coupled receptor bombesin receptor and appears to be involved in the response to oxidative stress [30]. As *rhodopsin* is also a GPCR, it is possible that the product of *CG4558* may be involved in facilitating the signaling through or impacting the stability of rhodopsin in the eye. This hypothesis is supported by another candidate, *Gycalpha99B*, which encodes a guanalyl cyclase enzyme most closely related to *GUCY1A1* and *GUCY1A2* in humans. Known to be important in the *rhodopsin* photoreceptor signaling pathway, mutation of *Gycalpha99B* results in disorganization of the ommatidia on its own [31]. Loss of this enzyme when *rhodopsin* signaling is already disrupted in the presence of the misfolding Rh1^G69D^ protein is, therefore, consistent with our observation of increased degeneration. 

*Nedd8* and *CG4306* may be linked to the ultimate cell fate decisions after activation of the unfolded protein response by mis-folding *rhodopsin*. *Nedd8* encodes a ubiquitin-like polypeptide important for protein degradation that is conserved in humans (*NEDD8*). Neddylation of proteins is linked to several processes, including signal transduction, cell cycle regulation, and protein ubiquitination and degradation [32]. ER-associated degradation of proteins (ERAD) is upregulated under conditions of ER stress similar to that induced by expression of the mis-folding Rh1^G69D^ protein [33]. Previous studies have demonstrated that other genes in the ERAD pathway can also modify this exact model of RP [34]. When ERAD and other recovery mechanisms for the cell fail, it will instead undergo apoptosis [33]. *CG4306 (GGCT)* encodes a γ-glutamylcylotransferase that induces apoptosis by stimulating the release of cytochrome c from the mitochondria [35,36]. This is achieved downstream of the JNK signaling pathway, which is itself stimulated downstream of the ER stress response [33,35]. Blocking the pathway at this point by reducing the expression of GGCT could prevent activation of cell death and prevent degeneration, as we observe in the correlation analysis (Figure 7) and in the model (Figure 12). 

In all cases, it is possible that the modifiers may be acting independently of the Rh1^G69D^ model to alter eye size. For example, *Nedd8* is required for the appropriate regulation of the *Drosophila* B-catenin gene *armadillo*, and mis-regulation of this process leads to abnormal eye development [37]. Future characterization of these modifier genes will explore whether the mechanism by which eye size is affected is specific to an interaction with RP disease pathways or whether it impacts eye development independently of these pathways.

## 6. Conclusions and Future Works

The clustering algorithm used in this study was able to identify 89 statistically significant genes with a notable correlation between mean eye size and genetic expression. Among the identified genes, seven of the top ten suspected genes with probable ties to RP were run through a validation study.

There are several ways this study can continue to expand from both computational and biological perspectives. Short-term examples include annotating suspected genes based on other variations of clustering attributes, such as eye size. Another example would be expanding the pool of shared strains and mean eye sizes beyond the Rh1^G69D^ and DGRP files. With more available data, silhouette analysis can be used more effectively to determine the recommended number of clusters to use rather than function as a guide for the algorithm.

More long-term goals include incorporating other clustering algorithms, such as DBSCAN and Gaussian mixtures. The process can also be modified to account for genetic mutations and fitness computations in the cluster outlier selection process [38]. This would require obtaining information on any DGRP chromosomes associated with an increase or decrease in eye size when mutated. Lastly, with a larger pool of data to be fed into an autonomous analytic model, supervised machine learning would become a viable option for gathering candidate modifiers for RP.

## Reference 

## Figures and Tables

**Table 1:** Rh1^G69D^ data excerpt.

Strain	Mean_Eye_Size
RAL021	19,976.8
RAL026	21,473.22222
RAL038	19,981.5
RAL040	16,992.9
RAL042	21,481.4
RAL045	18,578.88889
RAL049	16,939
RAL057	17,144.4
RAL059	20,975.36364
RAL069	21,309.9
RAL073	21,332.4
RAL075	18,672.2
RAL083	21,022.9
RAL085	20,442.5

**Table 2:** *Drosophila* Genetic Reference Panel (DGRP) expression data excerpt.

Gene	line_21:1	line_21:2	line_26:1	line_26:2
FBgn0000014	4.244723137096	4.216353087773	4.028685457103	3.965513773625
FBgn0000015	3.234859699465	3.199773952148	3.266073854988	3.514853683793
FBgn0000017	8.066864661954	7.962031504804	8.016965852717	8.081375653861
FBgn0000018	5.317033087996	5.268665082586	5.583749673928	4.949218486350
FBgn0000022	3.000683083262	3.000127343072	4.033542617316	3.364429304288
FBgn0000024	6.120670812586	6.023183171389	6.363472660596	6.839307459595
FBgn0000028	4.101309577739	4.050933403680	4.581349625692	4.276622648091
FBgn0000032	7.460913282329	7.686897989778	7.782455553083	7.635495635919
FBgn0000036	3.988090417266	3.789139102527	3.979189512126	3.953967140263

**Table 3:** Candidate modifiers of retinitis pigmentosa (RP). Top modifiers shared among all three correlation analyses. Closest human orthologues are listed.

FBGN_ID	Gene Symbol	Gene Name	Human Ortho.	Link to RP
FBgn0013972	CG1912	*Gycalpha99B*	*GUCY1A1; GUCY1A2*	Involved in phototaxis mediated by rhodopsin
FBgn0029914	CG4558	*CG4558*	*C6orf89*	Interacts with GPCR
FBgn0031176	FBgn0031176	*CG1678*	*WHE*	
FBgn0031267	CG13688	*lpk2*	*IPMK*	
FBgn0031885	CG13778	*Mnn1*	*MEN1*	Tumor suppressor involved in the stress response
FBgn0032040	CG13386	*CG13386*		
FBgn0032725	CG10679	*Nedd8*	*NEDD8*	Involved in protein ubiquitination and degradation
FBgn0036787	CG4306	*CG4306*	*GGCT*	Regulates apoptosis through the release of cytochrome c from the mitochondria
FBgn0053177	CG33177	*CG33177*	*MGST1*	Protects from oxidative stress at the ER membrane
FBgn0065057	CR33726	*scaRNA:MeU2-C28*	*snoRNA*	

## Data Availability

Genomic sequence and gene expression data for the DGRP is available at http://dgrp.gnets.ncsu.edu/ (accessed on 23 March 2020). Gene expression data were initially published in Huang et al. 2015. Eye size data are available as a supplementary file from Show et al. 2016. Code can be downloaded at: https://github.com/jamstutz92/research-paper-2021.git (accessed on 15 February 2022).
